# 

**Published:** 1906-05

**Authors:** 


THE AMERICAN

### Journal of Dental Science

Founded, Owned, Edited and Published by the Dental Profession of America.

THIRD SERIES.

VOLUME XXXVII. MAY, 1906. NO. 5.

EDITORS:

WM. GIRD BEECROFT, D. D. S., Madison, Wis. B. H. TEAGUE, D. D. S., Aiken, S. C

Published on the 10th day of each month.
Subscription Price, $1.00 per year. Foreign countries, $1.50 per year.

*Entered as Second-Class Matter, Madison, Wis.*

Address all communications, both business and editorial, to
The Dental Publishing Company, Madison, Wisconsin.

### SOCIETY ANNOUNCEMENTS.

Grand Reunion of the Alumni of the Philadelphia Dental College. Alumni day falls upon Thursday, May 31st, and will be celebrated by clinics, business meeting and a banquet or reception in the evening. Dr. Wheeler of New York will read the annual paper.

North Carolina Board of Dental Examiners. Meeting for the examination of applicants will be held at High Point, June 18th, 19th and 20th. Written examination in all regular college branches together with practical work in both operative and mechanical dentistry. Only graduates will be admitted to the examination and each applicant must bring his diploma.

R. H. Jones,
*Secretary.*

Winstom Salem, N. C.

The Southern Dental Society of the State of New Jersey will hold all day clinics May 16th and 17th and listen to illustrated lectures Wednesday evening, May 16th. The entire fifth floor of the Temple building, 413–415 Market St., has been provided for the occasion.

South Carolina State Dental Association will meet in annual session at Charleston on the Isle of Palms, June 26th, 27th, 28th and 29th. A cordial invitation is extended to all ethical dentists.

Maine Dental Society holds its forty-first annual meeting at Kineo House, Moosehead Lake, Maine, July 17th, 18th and 19th.

Kentucky State Dental Association. The time and place for the annual meeting of this association has been changed to Louisville, during home coming week, June 12th, 13th and 14th. Special railroad rates of one fare for the round trip can be obtained. All former residents of Kentucky in the profession are especially invited.

Kentucky State Board of Dental Examiners meets at Louisville, June 5th. All applicants will be examined in regular college branches. Practical work, gold and amalgam. One piece of metal work, materials, small instruments and engine must be furnished by the candidate. The fee is $20.00.

J. Richard Walton,
*President.*

The Masonic, Louisville, Ky.

